# Cecal microbiome composition and metabolic function in probiotic treated broilers

**DOI:** 10.1371/journal.pone.0225921

**Published:** 2020-06-03

**Authors:** Denise R. Rodrigues, Whitney Briggs, Audrey Duff, Kaylin Chasser, Raj Murugesan, Chasity Pender, Shelby Ramirez, Luis Valenzuela, Lisa Bielke

**Affiliations:** 1 Department of Animal Sciences, The Ohio State University, Columbus, Ohio, United States of America; 2 BIOMIN America Inc., Overland Park, Kansas, United States of America; 3 BIOMIN Holding GmbH, Getzersdorf, Austria; USDA-Agricultural Research Service, UNITED STATES

## Abstract

Probiotics have become increasingly popular in the poultry industry as a promising nutritional intervention to promote the modulation of intestinal microbial communities and their metabolic activities as a means of improving health and performance. This study aimed to determine the influence of different probiotic formulations on the taxonomic and metabolic profiling of cecal microbial communities, as well as to define associations between cecal microbiota and growth parameters in 21 and 42-day-old broilers. Probiotics investigated included a synbiotic (SYNBIO), a yeast-based probiotic (YEAST), and three single-strain formulations of spore-forming *Bacillus amyloliquefaciens* (SINGLE1), *B*. *subtilis* (SINGLE2) and *B*. *licheniformis* (SINGLE3). Dietary inclusion of SYNBIO, YEAST, SINGLE2, and SINGLE3 into the diets supported a significant stimulation of BW and BWG by 7 days of age. Besides, SYNBIO reduced the overall mortality rate by 42d (*p*<0.05). No significant variation was observed among different probiotic-based formulations for cecal microbiota composition. However, there was a treatment-specific effect on the metabolic profiles, with a particular beneficial metabolic adaptation by the microbiota when supplemented by SYNBIO and SINGLE2. Furthermore, the population of Lactobacillales was identified to be strongly associated with lower Enterobacteriales colonization, higher BW means, and lower mortality rate of growing broilers. Overall, the results emphasize that probiotic supplementation may enhance the microbial energy metabolism in the ceca of young broilers.

## Introduction

Worldwide, the decreased percentage of chickens treated with sub-therapeutic levels of antibiotics has attracted attention towards a better understanding of dietary alternatives as growth and health promoters. Among them, probiotics have been indicated as a promising nutritional intervention to manipulate the avian microbiome and its metabolites production [1–4]. Beneficial bacteria colonization of intestinal microbiota is essential for favoring host growth and health, while an unfavorable alteration of the commensal structure may promote enteric infections, thereby deteriorating welfare and the performance indicators of poultry production [5].

Probiotics have become increasingly popular across human medicine and livestock industry due to the following benefits in the host: stimulation of beneficial microbiota, reduction, and prevention of pathogen colonization, development of epithelial cell and immune system, and improvement in host energy intake [3,5–9]. Although several bacterial species and yeasts have been described as potential probiotic for broiler chickens; *Bacillus*, *Lactobacillus*, *Enterococcus*, *Bifidobacterium*, *Pediococcus*, and *Escherichia* are the most common bacterial genera used for probiotic formulations, whereas *Saccharomyces cerevisiae* is the most common yeast [5,7]. Some of the factors that have been claimed to be responsible for probiotic’s efficiency include the microbial viability in the gastrointestinal tract (GIT), the ability to adhere to epithelial cells and colonize the host GIT, capability to reproduce itself in the host, and production of essential metabolites [9,10].

Although the emergence of 16S rRNA sequencing and metagenomics pipelines has promoted deep insights into how intestinal microbiota is associated with health and disease, there is still a gap in knowledge concerning the effectiveness of probiotic supplementation in shaping GIT microbial communities and their metabolic activities. Accordingly, a comprehension of how the taxonomic profiling modulated by probiotics can affect the functional capabilities of microbes in the gut will improve our understanding of microbial metabolic activities and their role in poultry metabolism and health. Therefore, the primary aim of this study was to determine the effects of different probiotic formulations on the cecal microbial communities' composition and their potential metabolic functions, as well as to define associations between ceca microbial profile and growth parameters in broiler chickens.

## Material and methods

### Experimental design and dietary treatments

A total of 720 one-day-old Ross 708 male chicks were allocated to 6 treatments in a completely randomized design. The chicks were obtained from a commercial local hatchery. Eight replicates were assigned to each of the treatments with 15 birds per replicate. Treatments were based on supplemental diets including (1) basal diet without probiotics (CON); (2) Synbiotic (0.45 g/Kg; SYNBIO); (3) Yeast-based probiotic (1.12 g/Kg; YEAST); (4) Single-strain probiotic 1 (0.45 g/Kg; SINGLE1); (5) Single-strain probiotic 2 (0.27 g/Kg; SINGLE2) or (6) Single-strain probiotic 3 (0.45 g/Kg; SINGLE3).

The SYNBIO-based mixture was composed of 2 × 10^11^ CFU/g multi-species probiotic including *Lactobacillus reuteri*, *Enterococcus faecium*, *Bifidobacterium animalis*, *Pediococcus acidilactici*, and a prebiotic (fructooligosaccharide). The formulation YEAST was a probiotic-containing *Saccharomyces cerevisiae* (Moisture 11%, Crude fiber 25%). The single-strain probiotics were composed of spore-forming *Bacillus* spp. Formulation SINGLE1 contained 1.25 × 10^6^ CFU/g of *B*. *amyloliquefaciens*, while SINGLE2 comprised 10 billion spores/g of *B*. *subtilis*. Besides, each gram of the SINGLE3 contained 3.20 ×10^9^ CFU of *B*. *licheniformis*.

Birds were reared from 1 to 42d and housed in floor pens on fresh wood shavings litter with *ad libitum* access to a standard corn-soy diet and water (S1 Table) [11]. The feeding program consisted of 3 phases: starter (1-7d), grower (8-21d), and finisher (22-42d). Stater diets were in mash form, whereas the grower and finisher diets were pelleted. All experimental procedures were approved by the Ohio State University’s Institutional Animal Care and Use Committee (IACUC) number 2018A000000014.

### Growth performance

The birds were weighed individually weekly for the overall experimental period. Feed consumption for each pen was recorded by measuring feed residue on the same days as birds were weighed. Feed conversion ratio (FCR) was calculated as pen feed consumption divided by body weight gain per pen, corrected for mortality. Mortality was showed as cumulative mortality per treatment by 21 and 42 days of age.

### Sample collection and processing

We selected four birds per pen to investigate the intestinal microbiota composition of probiotic-treated broilers on days 21 and 42. Post-euthanasia, the samples from cecal contents were collected, immediately immersed in liquid nitrogen and kept frozen at -18°C until further DNA extraction. Cecal contents were weighed and mixed to create pooled samples from two birds (n = 16 per treatment for each time collection) for DNA extraction. Next, 0.3 g of the mixed digesta was added into a 2.0 mL screwcap microcentrifuge tube with 0.2 g of zirconia beads (0.1 mm). DNA was extracted from each sample, along with pure culture bacterial samples, using the protocol from Arthur et al. [12] with several modifications. In brief, phenol: chloroform: iso-amyl alcohol (25:24:1, 1 phase) was used for all DNA washings, during which the extraction sample supernatant was mixed with 500 μL of the phenol: chloroform: iso-amyl alcohol. After adding Buffer AL (Qiagen, Germantown, Maryland) and ethanol, samples were placed on to EconoSpin Silica Membrane Mini spin columns (Epoch Life Science Inc., Missouri City, TX, USA) and centrifuged (14,000 rpm at 21°C) for the same time durations rather than placed onto a vacuum manifold. After extractions were completed, DNA quality and quantity were measured using a Synergy HTX, Multi-Mode Reader (BioTek, Winooski, VT), and all samples were diluted to a concentration of 20 ng/μL for 16S rRNA sequencing analysis.

### 16S rRNA library preparation and sequencing methods

High quality RNase-treated genomic DNA was submitted to the Molecular and Cellular Imaging Center (MCIC, http://mcic.osu.edu/home) in Wooster, Ohio for library preparation. The DNA samples were quantified and normalized before library preparation. The V4 hypervariable region of the bacterial 16S rRNA gene was targeted in this study. To amplify and sequence the region of interest, we used primers that contain a heterogeneity spacer in line with the targeted sequence. Four sets of spacers of different lengths were used to compensate for the low nucleotide diversity of the amplicons; since accurate base-calling on Illumina platforms and generation of high-quality data requires sequence diversity at each nucleotide position before the clustering occurs. For the targeted region, we used 515F and 806R primers (515F: GTGYCAGCMGCCGCGGTAA, 806R: GGACTACHVGGGTWTCTAAT), which include degenerate bases for maximal inclusiveness [13].

Libraries were prepared in two rounds of PCR amplification. The first round amplified the locus of interest and added a portion of the Illumina adapter sequence, and the second round completed the Illumina adapter sequence which contained a unique dual combination of the Nextera indices for individual tagging of each sample. Twenty-five nanograms of each genomic DNA were used as input for the first PCR reaction and 3 uL of the clean PCR 1 product was used as input for the second PCR reaction. PCR amplifications were carried as follows: initial denaturation at 96°C for 3 min, followed by 25 (PCR 1) or 8 (PCR 2) cycles each of 96°C for 30 s, 55°C for 30 s and 72°C for 30 s, and a final extension at 72°C for 5 min. The PCR products were purified after each PCR amplification using the Agencourt AMPure XP beads (Beckman Coulter Life Sciences). All the steps for library preparation and cleaning were carried out on the epMotion5075 automated liquid handler (Eppendorf). The purified amplicon libraries were quantified and pooled at equimolar ratios before sequencing. The final pool was validated for size and absence of primer dimers on the TapeStation 4200 system (Agilent) and quantified using Qubit 2.0 fluorometer system (ThermoFisher Scientific).

The amplicon libraries were using the MiSeq sequencing platform (Illumina) at a final concentration of 14.3 pM. PhiX was mixed in with the pool of amplicon libraries for the sequencing run (expected at 20%). The run was clustered to a density of 771 k/mm2 and the libraries were sequenced using a 300PE MiSeq sequencing kit with the standard Illumina sequencing primers. Image analysis, base calling and initial data quality assessment were performed on the MiSeq instrument.

### Bioinformatics processing

A sequencing quality screen was performed to ensure high-quality sequences. Briefly stated, sequence quality was determined using the FASTQC and MultiQC toolkits [14]. Sequence reads exhibiting a quality score of lower than 20 were removed. Further, low complexity reads, those shorter than 200 bp in length, and mismatched primers were also eliminated. Additionally, reads exhibiting low sequence qualities on either end were trimmed.

Microbiome bioinformatics were performed with QIIME2 2019.7 [15]. The main analytical steps were as follows: firstly, reads were de-multiplexed and classified into their respective samples. Next, additional sequence quality control measures and feature table construction were performed by the DADA2 algorithm [16].

Taxonomy was assigned to amplicon sequence variants (ASVs) using the q2-feature-classifier plugin against the Greengenes 13_8 99% OTUs reference sequences database [17]. The resulting feature table was used to calculate phylum and order-level abundance infographics.

PICRUSt2 (Phylogenetic Investigation of Communities by Reconstruction of Unobserved States) was used for predicting the functional profiling of microbial communities in 21-days-old broilers based on the 16S rDNA sequences [18]. We used STAMP v2.1.3 (https://beikolab.cs.dal.ca/software/STAMP) software package for analyzing the metabolic potential of the microbial communities. The functional profiling was built based on the MetaCyc Metabolic Pathway Database [19].

The sequencing datasets for this study are available at Sequence Read Archive under BioProject accession number PRJNA578362.

### Statistical analysis

Body weight (BW), Feed intake (FI), BW gain (BWG), FCR and relative abundances of microbial communities were compared by ANOVA, followed by the Tukey post hoc test (*p* ≤0.05) using the JMP Pro13 Software (JMP Software, SAS Inc., 2018). For mortality, data were analyzed using the Chi-Square test (*p* ≤ 0.05) in SAS (SAS Inc., 2018). The statistical differences for microbial functions were performed by STAMP. Two-side Welch’s t-test was used for pairwise comparison of microbial pathways abundance against the control group *(p* ≤ 0.05). The Principal Component Analysis (PCA) was performed by applying ANOVA, followed by the Tukey-Kramer post hoc test (*p* ≤0.05) and Benjamin–Hochberg FDR for correction. Additionally, the Spearman's rank correlation coefficient (R) was applied to identify correlations between bacterial colonization patterns and performance parameters (R software version 3.4.1).

## Results

### Growth performance parameters

Dietary inclusion of SYNBIO, YEAST, SINGLE2, and SINGLE3 increased BW and BWG by 7 days of age compared to CON (*p<*0.05; Table 1).

**Table 1:** Performance parameters of broilers fed different probiotics from 1 to 42 days of age.

Days	CON	SYNBIO	YEAST	SINGLE1	SINGLE2	SINGLE3	*p*-value	SEM
	**Body Weight (Kg) ± SE**							
d0	0.36 ± 0.2	0.36 ± 0.2	0.37 ± 0.2	0.36 ± 0.2	0.36 ± 0.2	0.37 ± 0.2	0.402	0.09
d7	0.114 ± 2.2^c^	0.123 ± 1.8^a^^b^	0.124 ± 2.2^a^^b^	0.116 ± 2.1^b^^c^	0.123 ± 2.0^a^^b^	0.126 ± 1.9^a^	< .001	0.84
d14	0.330 ± 7.0^a^^b^	0.354. ± 5.3^a^^b^	0.355 ± 6.4^a^	0.329 ± 5.9^b^	0.348 ± 6.3^a^^b^	0.347 ± 6.2^a^^b^	0.005	2.57
d21	0.738 ± 14.1^a^^b^	0.779 ± 10.3^a^	0.774 ± 13.2^a^^b^	0.729 ± 11.8^b^	0.774 ± 12.0^a^^b^	0.764 ± 11.8^a^^b^	0.013	4.98
d28	1,335± 29.5	1,408 ± 20.7	1,384 ± 26.7	1,315 ± 26.7	1,392± 23.2	1,380 ± 26.7	0.084	10.45
d35	2,075 ± 42.2	2,159 ± 31.6	2,129 ± 36.1	2,031 ± 40.7	2,144 ± 38.2	2,156 ± 37.7	0.111	15.28
d42	2,983 ± 63.7	3,033 ± 46.7	2,981 ± 55.4	2,893 ± 61.2	3,051 ± 58.5	3,054 ± 55.1	0.343	22.76
	**Body Weight Gain (Kg) ± SE**							
d0-7	0.77 ± 2.1^c^	0.86 ± 1.7^a^^b^	0.87 ± 2.1^a^^b^	0.80 ± 2.0^b^^c^	0.86 ± 2.0^a^^b^	0.89 ± 1.9^a^	< .0001	0.82
d7-14	0.216 ± 5.2	0.231 ± 4.3	0.230 ± 5.0	0.213 ± 4.4	0.225 ± 4.8	0.221 ± 4.7	>0.05	1.95
d14-21	0.408 ± 8.0^a^^b^	0.424 ± 6.5^a^	0.412 ± 12.1^a^^b^	0.399 ± 7.6^b^	0.419 ± 10.1^a^^b^	0.417 ± 6.3^a^^b^	0.043	2.86
d21-28	0.601 ± 13.8^a^^b^	0.640 ± 10.6^a^	0.617 ± 19.0^a^^b^	0.576 ± 16.6^b^	0.620 ± 14.8^a^^b^	0.628 ± 12.3^a^^b^	0.025	5.01
d28-35	0.735 ± 18.2	0.770 ± 14.7	0.738 ± 15.8	0.707 ± 21.9	0.758 ± 24.2	0.774 ± 15.3	0.102	6.62
d35-42	0.899 ± 23.8	0.911 ± 37.8	0.851 ± 28.9	0.886 ± 25.2	0.907 ± 39.0	0.901 ± 23.9	0.757	12.49
	**Feed Intake (Kg) ± SE**							
d0-7	0.119 ± 3.9^b^	0.135 ± 2.0^a^	0.131 ± 4.8^a^^b^	0.130 ± 2.5^a^^b^	0.137 ± 3.5^a^	0.132 ± 2.3^a^^b^	0.016	0.03
d7-14	0.273 ± 12.1	0.259 ± 8.6	0.268 ± 9.1	0.261 ± 10.5	0.257 ± 6.5	0.264 ± 5.8	0.85	0.02
d14-21	0.534 ± 18.2	0.595 ± 11.3	0.602 ± 32.4	0.551 ± 14.5	0.627 ± 30.2	0.583 ± 18.5	0.084	0.02
d21-28	0.569 ± 28.7	0.616 ± 16.6	0.585 ± 13.7	0.530 ± 32.8	0.576 ± 17.3	0.605 ± 17.8	0.104	0.02
d28-35	0.749 ± 33.1^a^^b^	0.792 ± 21.1^a^	0.774 ± 13.4^a^^b^	0.681 ± 36.2^b^	0.728 ± 25.6^a^^b^	0.828 ± 14.7^a^	0.003	0.02
d35-42	0.971 ± 44.1	0.980 ± 39.0	0.906 ± 14.8	0.880 ± 41.3	0.934 ± 30.7	0.990 ± 30.7	0.162	0.02
	**Feed Conversion Ratio (g/g) ± SE**							
d0-7	1.62 ± 0.09	1.61 ± 0.07	1.52 ± 0.08	1.64 ± 0.05	1.62 ± 0.08	1.50 ± 0.07	0.666	0.03
d7-14	1.27 ± 0.06	1.14 ± 0.04	1.18 ± 0.03	1.20 ± 0.05	1.17 ± 0.04	1.20 ± 0.03	0.511	0.02
d14-21	1.33 ± 0.01	1.43 ± 0.03	1.45 ± 0.06	1.41 ± 0.03	1.53 ± 0.07	1.40 ± 0.03	0.154	0.02
d21-28	1.52 ± 0.04	1.50 ± 0.02	1.50 ± 0.04	1.52 ± 0.06	1.48 ± 0.04	1.47 ± 0.05	0.945	0.02
d28-35	1.66 ± 0.04	1.63 ± 0.06	1.66 ± 0.03	1.63 ± 0.03	1.61 ± 0.06	1.68 ± 0.07	0.949	0.02
d35-42	1.83 ± 0.06	1.79 ± 0.06	1.76 ± 0.06	1.74 ± 0.05	1.81 ± 0.06	1.76 ± 0.03	0.891	0.02
d0-42	1.85 ± 0.06	1.85 ± 0.03	1.86 ± 0.04	1.90 ± 0.07	1.92 ± 0.09	1.80 ± 0.05	0.791	0.02
	**Cumulative mortality (mortality/ birds per treatment)**							
d0-21	11/120^a^	2/120^b^	3/120^b^	9/120^a^	5/120^a^	9/120^a^	< .001	1.50
d0-42	21/120^a^	10/120^b^	15/120^a^	20/120^a^	18/120^a^	15/120^a^	0.034	1.64

^a-c^ Different letters in the same row indicate statistical differences (*p*<0.05). Broilers fed basal diet without probiotics (CON), synbiotic (SYNBIO), yeast-based probiotic (YEAST), or single-strain formulations composed of *Bacillus amyloliquefaciens* (SINGLE1), *B*. *subtilis* (SINGLE2), and *B*. *licheniformis* (SINGLE3).

In addition, broilers fed SYNBIO had better BWG (*p<*0.05) than SINGLE1 from day 14 to 28. No significant differences were found in the BW or BWG means on days 35 and 42. Supplementation of SYNBIO and SINGLE2 in the diet increased (*p*<0.05) FI from day 1 to 7 (Table 1). In the period from day 28 to 35, the FI of birds fed SINGLE1 was statistically reduced. Furthermore, no significant effect of probiotic supplementation was observed in FCR during the overall experimental period.

There was a lower cumulative mortality rate in SYNBIO and YEAST treated birds at 21 days of age. On day 42, dietary inclusion of SYNBIO significantly reduced the overall rate of mortality (*p* = 0.03; Table 1).

### Microbiota composition

A total of 5,348,269 16S rRNA sequence reads were obtained. The number of sequence reads of overall samples ranged from 13,545 to 60,125 with a mean of 27,855.82. In order to assess the impact of different probiotic supplementation on cecal bacterial populations, the 16S-derived microbial community was analyzed at the taxonomic rankings of phylum and order levels.

**Fig 1:**
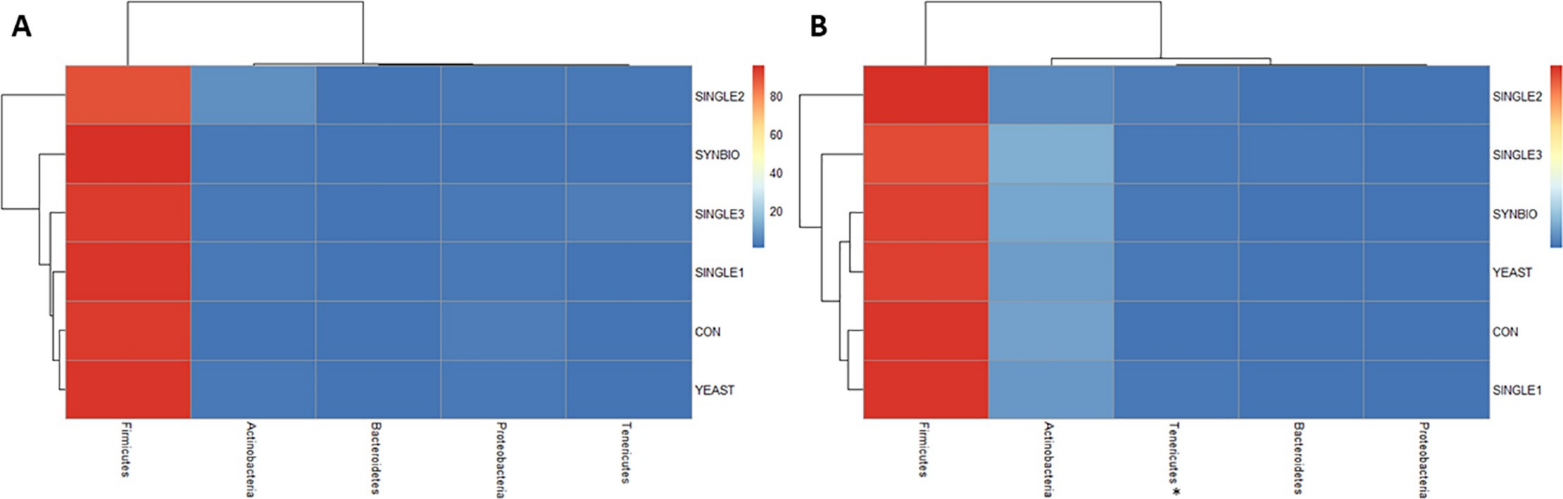
Cecal bacterial abundance at phylum-level of broilers fed different probiotics by 21 and 42 days of age. (A) Heatmap plot represents the composition of cecal microbiota from broilers fed basal diet without probiotics (CON), synbiotic (SYNBIO), yeast-based probiotic (YEAST), or single-strain formulations composed of *Bacillus amyloliquefaciens* (SINGLE1), *B*. *subtilis* (SINGLE2), and *B*. *licheniformis* (SINGLE3) by 21 and (B) 42 days of age. Hierarchical clustering in the rows is based on the composition similarity between treatments, while that in the columns is based on the microbial relative abundance's closeness. Statistical differences (*p*<0.05) between groups were reported for each bacterial population (*).

Similar to many microbiome previous studies, the assigned taxonomic profiles at the bacterial phylum showed that the dominant cecal populations at 21 days of age were Firmicutes (89.38–95.43%), Actinobacteria (1.29–6.25%), and Proteobacteria (0.94–2.85%). By 42 days of age, there was a minor change in the cecal microbial colonization pattern, in which the proportions of Firmicutes (84.53–89.25%) and Proteobacteria (0.15–0.25%) in the ceca were reduced, whereas Actinobacteria relative abundance (5.44–12.33%) increased over time. It may be observed that the Tenericutes population was greatly enhanced in YEAST, SINGLE1, and SINGLE2 compared with the CON group (*p*<0.05, Fig 1, S2 Table).

**Fig 2:**
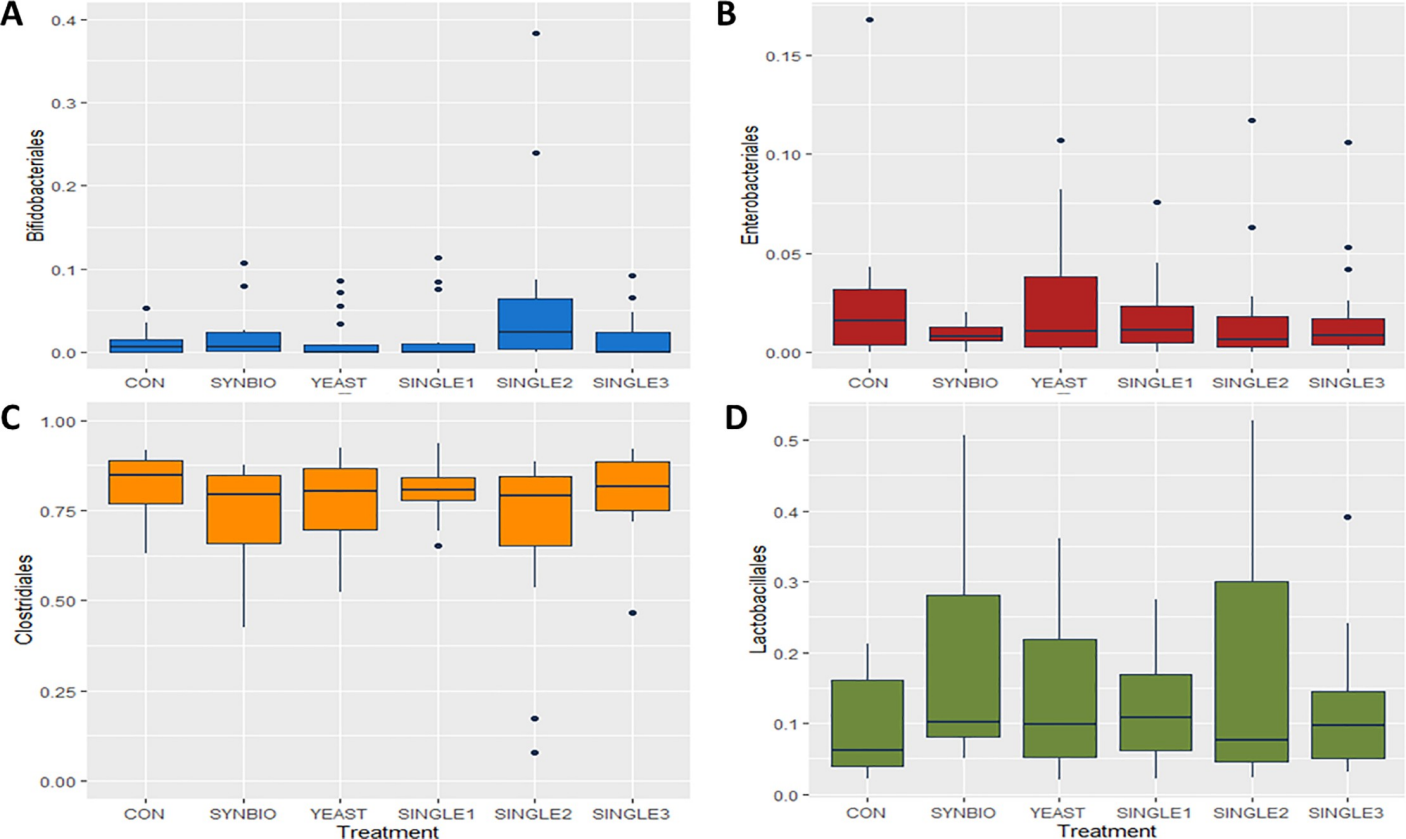
Microbial composition in the ceca digesta of 21-day-old broilers. Box plots show the relative abundance of the top four order-level bacterial population found in the ceca in broilers, including (A) Bifidobacteriales, (B) Enterobacteriales, (C) Clostridiales, and (D) Lactobacillales.

By 21 days of age, the microbiota composition was not significantly affected by the supplementation of probiotics. It was noted that Bifidobacteriales and Lactobacillales contributed most to the numerical differences in the microbial composition. Bifidobacteriales had the highest population in SINGLE2, whereas supplementation of SYNBIO increased the relative abundance of Lactobacillales (*p*>0.05, Fig 2)

**Fig 3:**
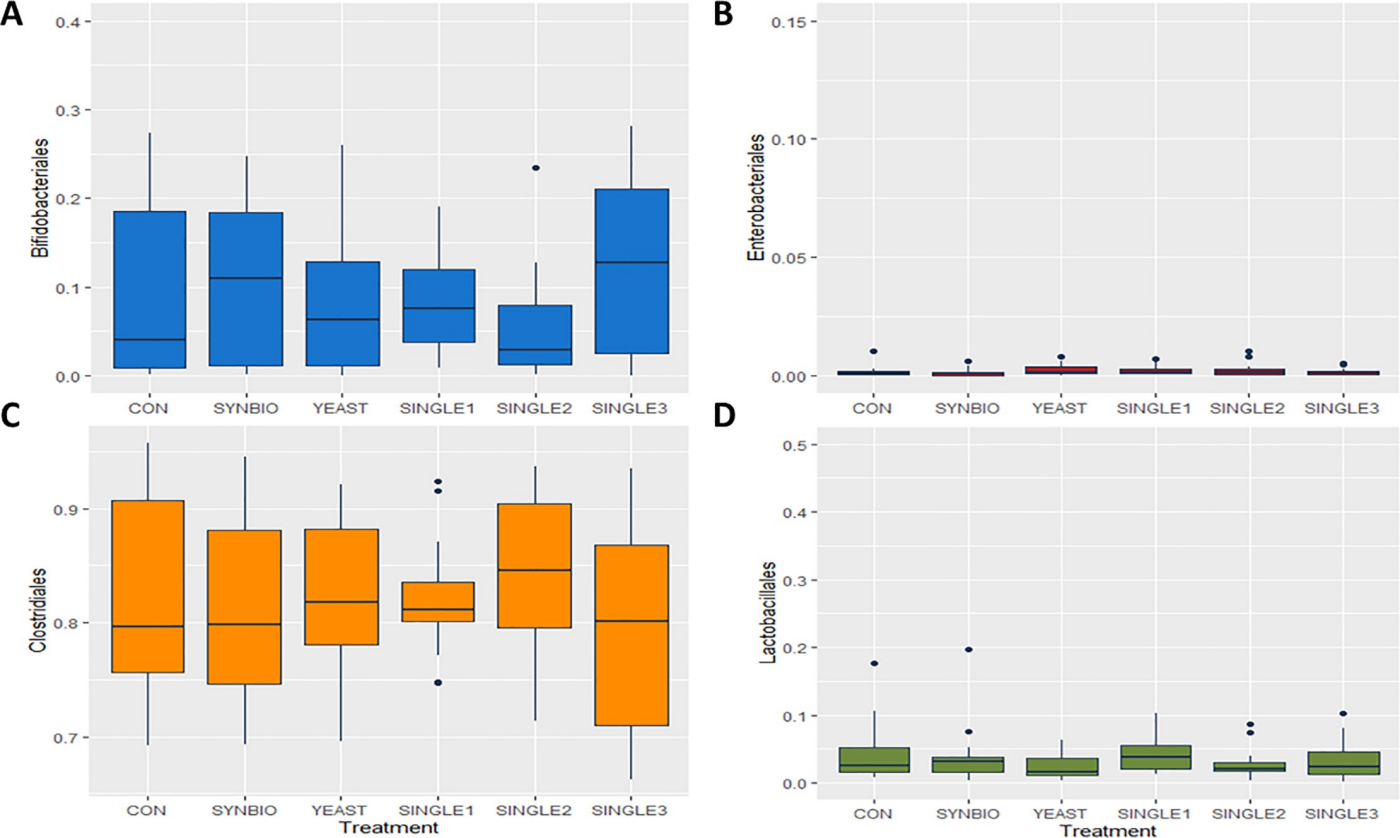
Order-level taxonomic distribution among samples from cecal contents of 42-day-old broilers. Box plots represent the mean relative percentage of each bacterial population within samples from broilers treated with a basal diet without probiotics (CON), synbiotic (SYNBIO), yeast-based probiotic (YEAST), or single-strain formulations composed of *Bacillus amyloliquefaciens* (SINGLE1), *B*. *subtilis* (SINGLE2), and *B*. *licheniformis* (SINGLE3). (A) Bifidobacteriales, (B) Enterobacteriales, (C) Clostridiales, and (D) Lactobacillales.

Dietary treatments had minimal effects on cecal microbiota by 42 days of age (Fig 3). In all treatments, the microbial composition was dominated by Clostridiales, while Enterobacteriales relative abundance reached a downward of 1%.

### Microbiota functional profiling

**Fig 4:**
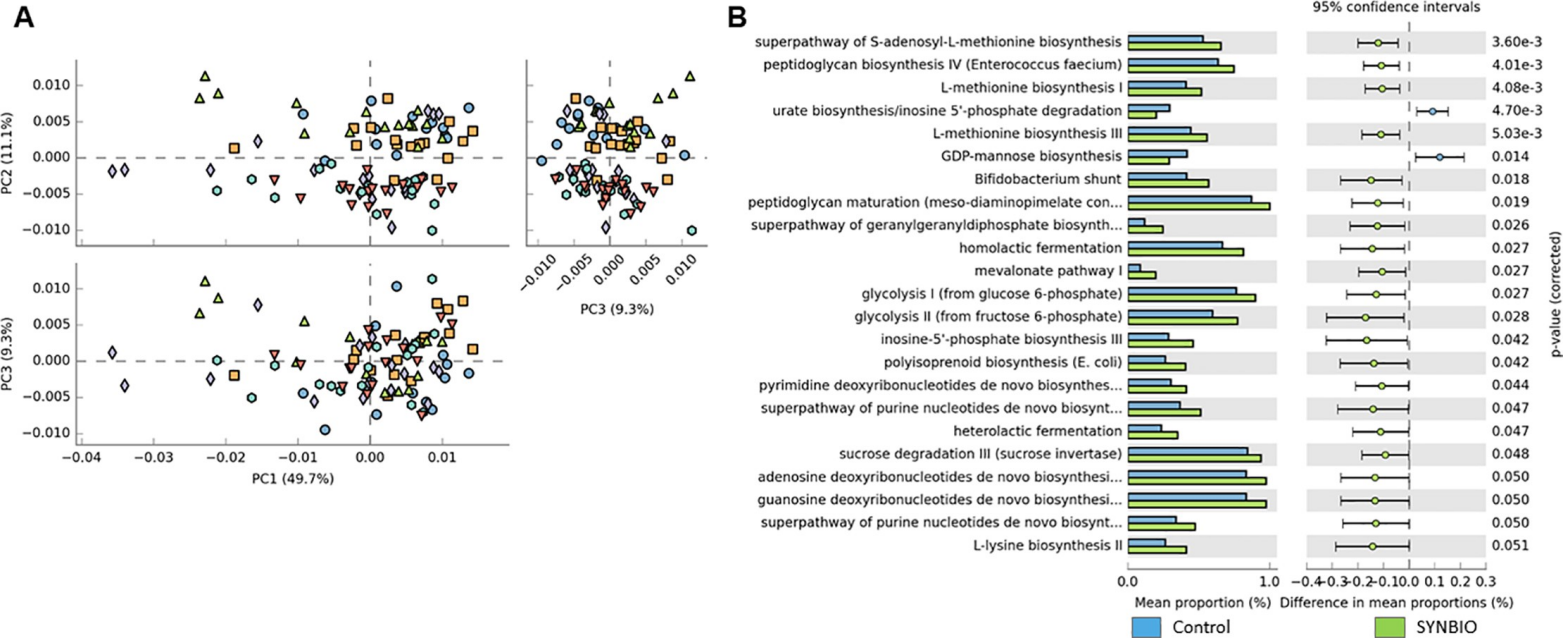
Predicted metabolic functions in probiotic treated broilers. (**A**) Principal component analyses (PCA) plots represent the potential metabolic functions of microbiota from broilers treated with a basal diet without probiotics (CON; blue sphere), synbiotic (SYNBIO; green triangle), yeast-based probiotic (YEAST; turquoise sphere), or single-strain formulations composed of *Bacillus amyloliquefaciens* (SINGLE1; dark orange down-pointing triangle), *B*. *subtilis* (SINGLE2; purple diamond), and *B*. *licheniformis* (SINGLE3; orange square). (**B**) Column bar graph showing the predicted metabolic pathways in SYNBIO in comparison with CON group.

We adopted PICRUSt2 to predict the functional profiling from 16S rRNA sequences with the purpose of describing the metabolic potential of the intestinal microbial community associated with different probiotic supplementation. In view of the most changes of microbiota composition were found in young broilers, we performed the metabolic profiling only from 21-days-old dataset. The PCA plots were determined to evaluate the similarity of the microbial functions between the probiotic and control treatments. PCA analyses did not reveal an evident clustering among the samples (Fig 4A).

**Fig 5:**
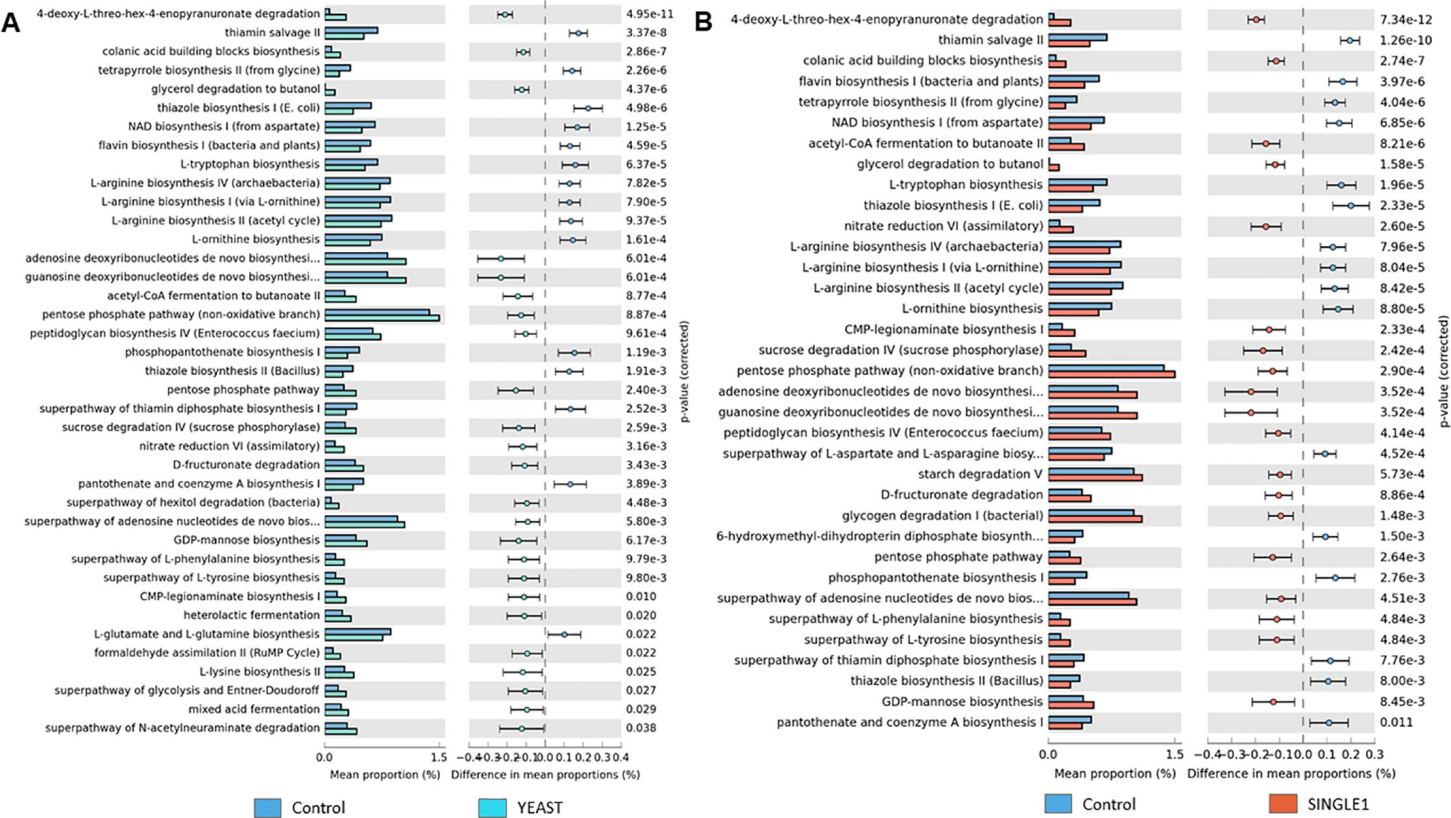
The abundance of metabolic pathways. (**A**) Predicted MetaCyc pathways in microbial communities from broilers supplemented with a yeast-based probiotic (YEAST) and (**B**) *Bacillus amyloliquefaciens* (SINGLE1) related to a basal diet without probiotics (CON).

**Fig 6:**
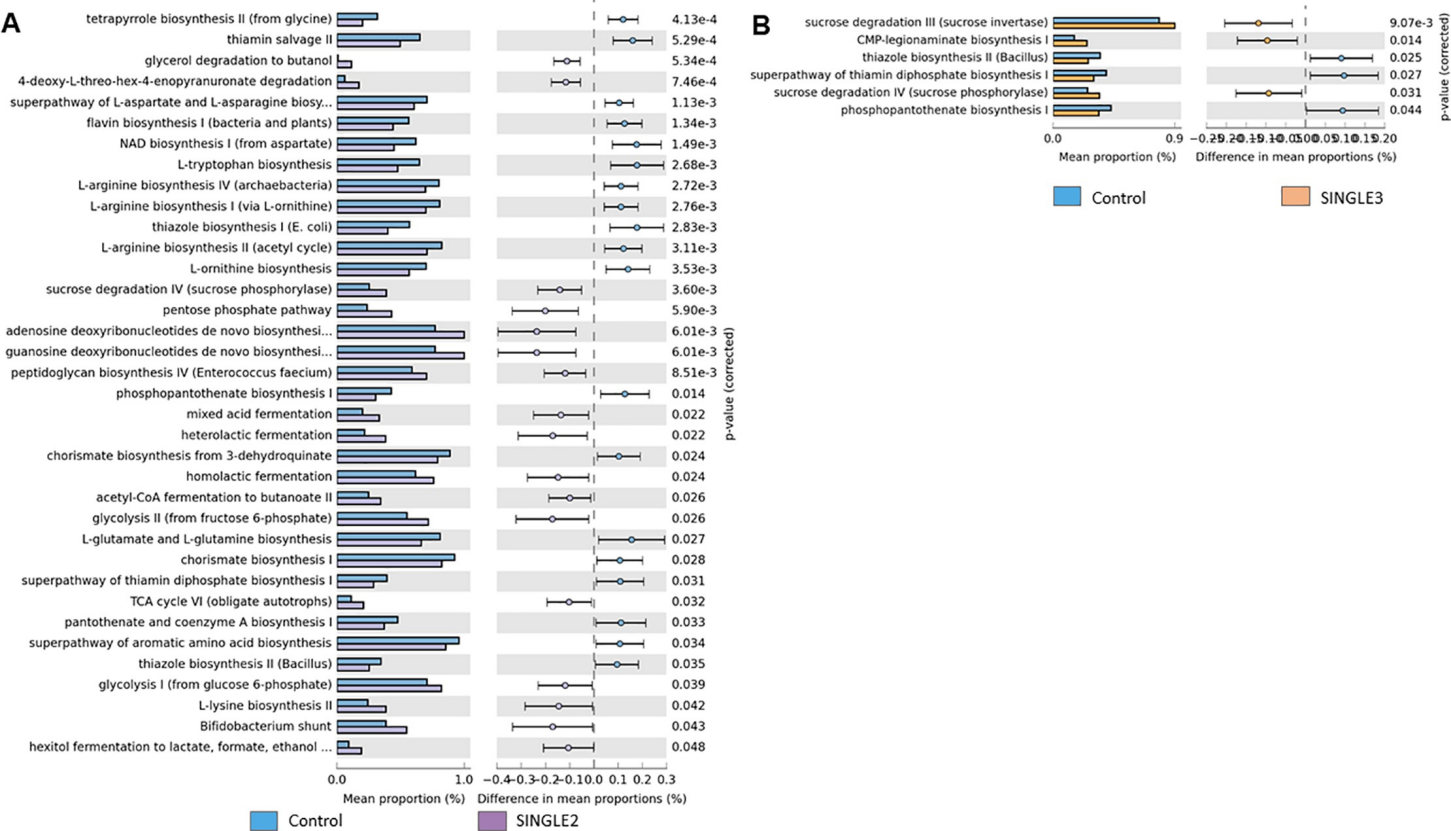
Functional annotation based on MetaCyc pathways. (**A**) Predicted metabolic pathways in cecal microbiota from broilers supplemented with a *Bacillus subtilis* (SINGLE2) and (**B**) *B*. *licheniformis* (SINGLE3) compared to a basal diet without probiotics (CON).

Nevertheless, based on the abundance profiling of microbial pathways, there were substantial differences (Two-side Welch’s t-test; *p*<0.05) between the predicted cecal microbial metabolic activities in probiotic treatments compared to CON (Figs 4B, 5 and 6).

Several core microbial pathways related to the amino acid, carbohydrate, nucleoside and nucleotide biosynthesis, as well as generation of precursor metabolite and energy were enriched with probiotic supplementation. To better understand the influence of different probiotic formulations on the metabolic functions of cecal microbiota, the enriched probiotic pathways were further described in a child class hierarchy (ontology) based on their biological functions, and on the classes of metabolites that they produce and/or consume (S3 Table).

The pairwise comparison of microbial pathways abundance revealed that in all probiotic treatments there was an enhancement of pathways related to energy metabolism. Pathway such as glycolysis was increased in SYNBIO and SINGLE2. Carbohydrate degradation and fermentation as sucrose, D-fructuronate, hexitol, likewise glycogen pathways, were enriched in SYNBIO, YEAST, SINGLE1, SINGLE2, and SINGLE3 suggesting an increased metabolic activity of energy sources in these treatments.

In addition to the enrichment of core metabolic processes described in Fig 4A, pathways related to biosynthesis and maturation of bacterial cell structure, carbohydrate degradation by Bifidobacterium, vitamin biosynthesis, lactic acid fermentation via homolactic, and heterolatic fermentation were enriched in SYNBIO. Whereas, the supplementation of YEAST overrepresented functional categories associated with biosynthesis of extracellular polysaccharide found in *Enterobacteriaceae*, biosynthesis of components from S-layer (Gram-positive bacteria) and lipopolysaccharides (LPS; Gram-negative), L-1,2—propanediol degradation, fermentation to short-chain fatty acids including heterolatic fermentation and biosynthesis of legionaminic acid, which can be identified as a virulence-associated cell-surface glycoconjugate in pathogenic bacteria [20] (Fig 5A). Metabolic activities enriched in SINGLE1 were also associated with L-1,2—propanediol degradation, biosynthesis of components from S-layer, and LPS, an *Enterobacteriaceae* extracellular polysaccharide, Gram-positive *Enterococci* cell structure and legionaminic acid (Fig 5B). In SINGLE2, the enriched predicted functions included biosynthesis of Gram-positive *Enterococci* cell structure, lactic acid fermentation, and carbohydrate degradation by Bifidobacterium (Fig 6A). Finally, only a few metabolic pathways were enhanced following the supplementation of SINGLE3 (Fig 6B). In addition to the attributes related to carbohydrate degradation, there was a predicted enrichment in the biosynthesis of legionaminic acid.

### Correlation between microbiota composition and performance parameters

**Fig 7:**
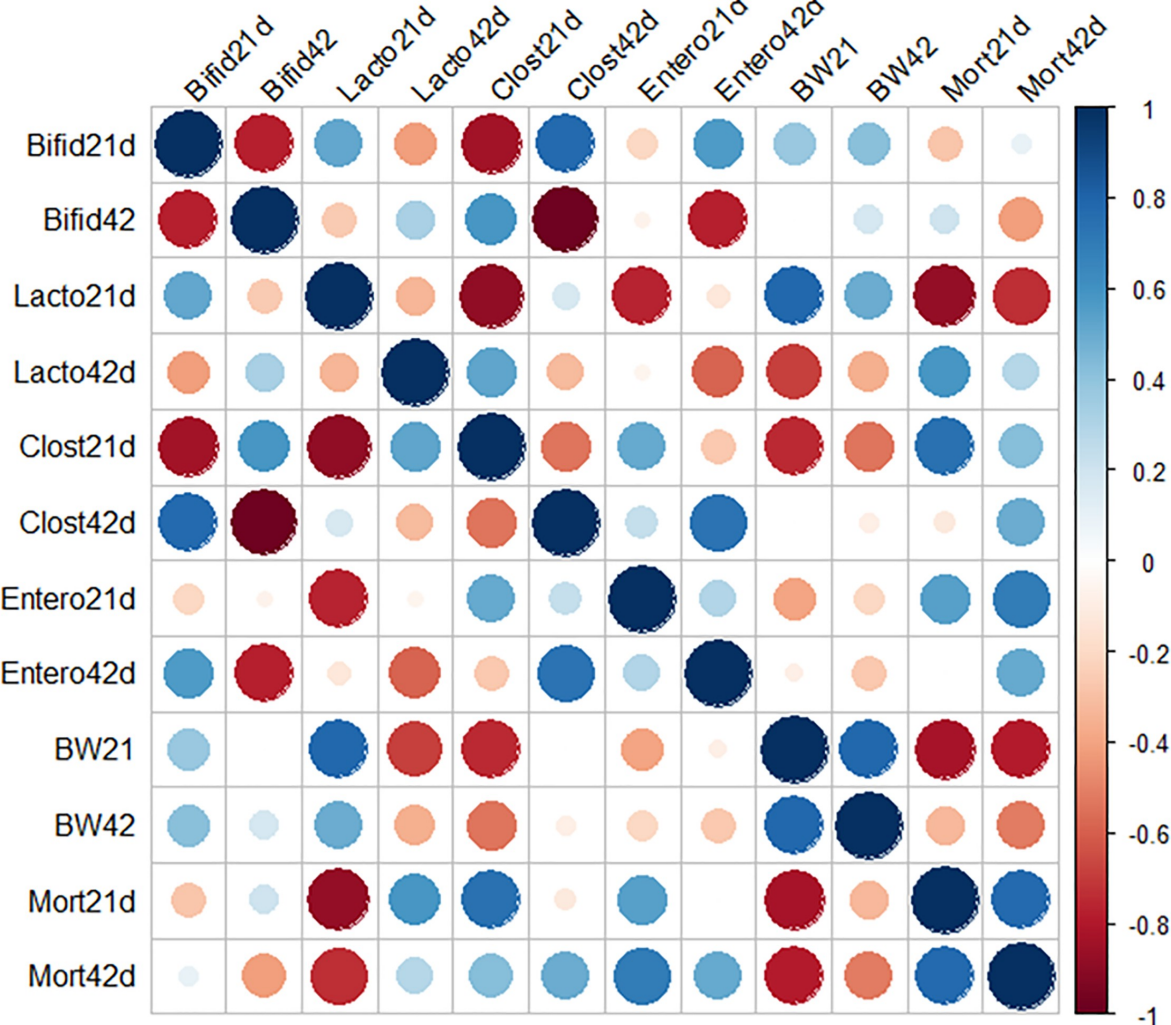
Spearman’s rank correlation matrix of the dominant microbial populations and growth performance parameters. (A) Large circles indicate strong correlations. The colors of the scale bar denote the nature of the correlation with 1 indicating a perfect positive correlation (dark blue) and -1 indicating perfect negative correlation (dark red).

To further analyze the associations between the cecal microbiome composition and host performance parameters, we conducted Spearman’s correlation linking the four discrepant order-level microbial taxa, BW means, and mortality rate by 21 and 42 days of age. The Spearman's rank correlation showed that abundances of Lactobacillales were negatively associated with Clostridiales by 21 days of age (Fig 7, S4 Table).

Another interesting interaction between components of microbiota was a negative correlation among Enterobacteriales and Lactobacillales. Likewise, the BW mean at 21 days of age positively impacted BW at 42 days of age, and negatively influenced the mortality rate at both ages. Additionally, we observed a strong positive correlation between the cecal Lactobacillales population and BW mean (R = 0.94, *p* = 0.048; Fig 7), whereas Lactobacillales' relative abundance was negatively associated with the mortality rate (R = -0.93, *p* = 0.007) on day 21. These results indicate that a higher population of Lactobacillales in the ceca may be a marker of better performance for young broilers (21 days of age).

We also found significant associations within the Clostridiales cecal population and performance parameters in 21-day-old broilers. Clostridiales was negatively related to BW (R = -0.89, *p* = 0.01; Fig 7) and positively associated with mortality by 21 days of age. Interestingly, the greatest correlation was only identified at an earlier age.

## Discussion

This study was conducted in order to gain a better comprehension of how different probiotic formulations could modulate the microbial taxonomic profiles and the metabolic activities of microbial communities in the ceca of broilers. Our findings indicate that the tested probiotic formulations neither elicit significant changes in the cecal microbiota populations nor altered growth parameters. Despite the cecal taxonomic similarities at order level by 21 days of age, there were differences in the predicted metabolic activities indicating that probiotic supplementation may play an important role in the microbial energy metabolism.

Probiotics seemed to have the greatest effect during the initial development of the microbiota [21]. As a consequence of limited contact with the hens' microbiota, the assembly of the intestinal microbiome of the newly hatched chicks is predominantly influenced by the hatchery and farm environment [22–24]. Thus, an immediate supplementation of probiotics post-hatch is more important in avian species than in other animals [7]. The early exposure to microbial preparations has been identified as an approach to modulate the microbiota towards beneficial bacterial growth [3,4] and pathogen colonization reduction [25]. Additionally, supplementation of probiotics has been successfully linked to GIT development by stimulating the growth of villus surface area [26–28]. Other probiotic action mechanisms include maturation of immune system, improvement of gut barrier function, and the presence of highly competitive microbial communities, which can lead exclusion of pathogenic bacteria through competitive exclusion [3,5–9].

The addition of SYNBIO, YEAST, SINGLE2, and SINGLE3 into the diets supported a significant stimulation of BW and BWG by 7 days of age. During the grower and finisher phase, there were no significant differences in BW, BWG, and FCR when compared with CON group. Significant differences were observed on day 14, in which broilers fed YEAST had higher BW than SINGLE1. Similarly, the dietary addition of SYNBIO outperformed SINGLE1 birds and had the greatest BW and BWG from day 14 to 21. In contrast with our results, supplementation of live yeast, yeast cultures, or yeast cell wall products was shown to have positive effects on animal performance [29–32]. Yalçın et al. [31] showed that *Saccharomyces cerevisiae* supplementation improved weight gain during the starter period of broiler chickens, although there were no effects on final weight (42d). Improvements in performance in SYNBIO supplemented broilers were previously reported by Eckert et al. [33]. Enhanced performance promoted by synbiotic products may be related to the improvement in nutrient absorption, reduction of pathogens colonization, and stimulation of the immune response [27,33,34].

The results here showed that the taxon abundances were similar between the probiotic treatments. However, based on the microbial pathway's abundance, there was a treatment-specific effect on the metabolic function showing a particular beneficial metabolic adaptation by the microbiota when supplemented by specific formulation such as SYNBIO and SINGLE2. This divergence between composition and functional profiling could be attributed to the differences in databases used to analyze the data, which could be considered a limitation of this study. On one side, the16S rRNA amplicon sequencing has considerably improved our understanding of how the intestinal microbiota and its metabolites are associated with health and disease. On the other side, this technique has a restricted ability to address sequences to deep taxonomic levels, such as genus and species. This fact may be important due to bacterial communities from the same order or even from the same species can have substantially divergent metabolic capabilities [35].

Our findings revealed enrichment of metabolic activities related to energy metabolism following probiotic supplementation at 21 days of age. It is well known that the dense population of microbial communities in the GIT ferment carbohydrates, as non-digestible carbohydrates, into short-chain fatty acids (SCFAs) and lactate as a means of producing ATP. Better regulation of the production of these energetic metabolites by commensal and probiotic bacteria may provide higher host energy intake [36]. Of relevance, PICRUSt2 results predicted that cecal microbiota in SYNBIO was also involved in vitamin biosynthesis. In fact, some *Lactobacillu*s strains have been reported by their capacity to produce B group vitamins [36]. The vitamins are acquired by the host through the diet and from gut microbial *de novo* synthesis [37], suggesting that supplementation of SYNBIO might perform a function on vitamin producing-microorganisms. Furthermore, the addition of SYNBIO in the feed resulted in an overabundance of lactic acid hetero- and homo-fermentative pathways. The lactic acid bacteria (LAB) produce lactate as the major end product of the fermentation of the carbohydrates [19]. In the heterofermentative pathway, in addition to lactate, substantial quantities of volatile acids and carbon dioxide are produced. While the homolactic fermentation, the LAB convert carbohydrates essentially into lactate [19]. This overabundance of lactic acid -fermentative pathways may be supported by our taxonomic results, in which SYNBIO increased the population of LAB (p>0.05). It is a widely held view that the addition of prebiotics into the mixture may support the growth and activity of the probiotic and GIT beneficial bacteria [38]. Similar results were found in layers, in which the addition of SYNBIO in the feed increased the relative abundance of LAB in ceca showing that the supplemented strains survived and colonized the GIT [21].

Interestingly, the CMP-legionaminate biosynthesis pathway was predicted to be enriched in YEAST, SINGLE1, and SINGLE3. The legionaminic acid is part of the diverse sialic acid family of α-keto sugars, which can be found as a virulence-associated cell-surface glycoconjugate in pathogenic Gram-negative bacteria [20]. Other enriched pathways in YEAST and SINGLE1 associated with Gram-negative colonization is the colanic acid building blocks biosynthesis and L-1,2—propanediol degradation [19]. It is worth highlighting that these chickens were under experimental conditions without a pathogen challenge or stress induction. Conversely, in SYBIO and SINGLE2 there was potential evidence of beneficial colonization by the identification of peptidoglycan biosynthesis IV (*Enterococcus faecium*) and Bifidobacterium shunt pathways. The Bifidobacterium shunt is described as a unique pathway used by Bifidobacterium for hexose catabolism [19].

We further looked for associations between the cecal predominant microbial signature and indicators of growth parameters. Lactobacillales population in ceca was positively correlated to BW on day 21 and negatively associated with mortality rate. The association between improved weight gain and LAB has also been observed by Yan et al. [39] and De Cesare et al. [40]. Hence, LAB may be able to enhance the energy and mineral recovery from nutrients; its higher intestinal colonization results in a better digestive efficiency [37,41]. Based on this correlative evidence, it is tempting to speculate that the supplementation of SYNBIO may have optimized a metabolic resource allocation to emerge a LAB population at 21 days of age, influencing the elevated survival of broilers found in this treatment. Future work with metabolomic analyses should be conducted to validate the metabolites associated with probiotic supplementation outcomes.

Spearman's correlation analyses also revealed that the Clostridiales was negatively related to BW by 21 days of age. Clostridiales population was the dominant order accounting for almost 83% of the entire cecal microbiota among treatments. Although Clostridiales members are known as the main responsible for short-chain fatty acid metabolism in chicken cecum [42], obtaining insights into how higher diversity and colonization of other bacterial communities in ceca can influence on the metabolic activities may have important implications for selecting probiotic formulations.

Overall, the results suggest that the supplementation of probiotics was not related to altered taxonomic profiling nor improved performance parameters in 21 and 42 day-old-broilers. Despite the similarity in microbial relative abundances at 21 days of age, there was a treatment-specific effect on the microbial metabolic functions indicating an enhancement of energy metabolism within probiotic treatments. The prediction of microbial metabolic activities using the 16S rRNA approach has limitations but continues being promising in the poultry industry. A future study exploring the combination of 16S rRNA sequencing and metagenomics, as well as metatranscriptomics and metabolomics, will provide more meaningful findings on the microbial metabolic activities and are urgently needed.

## Supporting information

S1 Table Composition of basal diet. (XLSX)

S2 Table Cecal bacterial abundance at phylum-level of broilers fed different probiotics by 21 and 42 days of age. (XLSX)

S3 Table List of enriched MetaCyc pathways ontology in the probiotic treatments in comparison with control group. (XLSX)

S4 Table Spearman’s rank correlation matrix of the dominant microbial populations and growth performance parameters. (XLSX)
